# Confocal Fluorescence Microscopy Investigation for the Existence of Subdomains within Protein Storage Vacuoles in Soybean Cotyledons

**DOI:** 10.3390/ijms23073664

**Published:** 2022-03-27

**Authors:** Hari B. Krishnan, Alexander Jurkevich

**Affiliations:** 1 Plant Genetics Research Unit, US Department of Agriculture-Agricultural Research Service, Columbia, MO 65211, USA; 2 Division of Plant Science and Technology, University of Missouri, Columbia, MO 65211, USA; 3 Advanced Light Microscopy Core, Christopher S. Bond Life Sciences Center, University of Missouri, Columbia, MO 65211, USA; jurkevica@missouri.edu

**Keywords:** confocal fluorescent microscopy, immunofluorescence localization, protein storage vacuoles, glycinin, β-conglycinin

## Abstract

In legumes, the seed storage proteins accumulate within specialized organelles called protein storage vacuoles (PSVs). In several plant species, PSVs are differentiated into subdomains that accumulate different kinds of proteins. Even though the existence of subdomains is common in cereals and legumes, it has not been reported in soybean PSVs. The two most abundant seed proteins of soybean, 7S and 11S globulins, have different temporal accumulation patterns and exhibit considerable solubility differences that could result in differential accretion of these proteins within the PSVs. Here, we employed confocal fluorescent microscopy to examine the presence or absence of subdomains within the soybean PSVs. Eosin-stained sections of FAA-fixed paraffin embedded soybean seeds, when viewed by confocal fluorescence microscopy, revealed the presence of intricate subdomains within the PSVs. However, fluorescence immunolabeling studies demonstrated that the 7S and 11S globulins were evenly distributed within the PSVs and failed to corroborate the existence of subdomains within the PSVs. Similarly, confocal scanning microscopy examination of free-hand, vibratome and cryostat sections also failed to demonstrate the existence of subdomains within PSVs. The subdomains, which were prominently seen in PSVs of FAA-fixed soybean seeds, were not observed when the seeds were fixed either in glutaraldehyde/paraformaldehyde or glutaraldehyde. Our studies demonstrate that the apparent subdomains observed in FAA-fixed seeds may be a fixation artifact.

## 1. Introduction

Soybean is a major source of protein and oil throughout the world. The seeds of soybean contain approximately 40% protein and 20% oil. The two most abundant seed storage proteins of soybean are the 11S glycinin and 7S β-conglycinin [1,2]. Together, these two groups of storage proteins account for about 70% of the total seed protein content of the seed. Because of their abundance, they not only influence the nutritive value but also affect the processing and utilization of food products derived from soybean. Glycinin, a hexameric protein, is composed of six nonrandomly paired acidic and basic peptides. The acidic peptides have molecular weights of approximately 44, 37, and 10 kDa, while the basic peptides exhibit molecular weights of 20–24 kDa [1]. Glycinins are synthesized as a larger precursor protein, which is post-translationally cleaved into acidic and basic subunits that are held together by a disulfide bond [3]. Glycinins are encoded by at least five gene family members and two pseudogenes [4,5]. The other abundant seed protein, the β-conglycinins, are glycoproteins and are composed of α´, α and β subunits [6]. Even though earlier studies claim that the β-conglycinins are encoded by multigene families [7], a recent study has shown that β-conglycinins are encoded by a relatively few genes [8].

Soybean storage proteins are synthesized during seed development and are under strict temporal and spatial regulation. The accumulation of mRNA encoding the different glycinin genes starts during early seed development, peaks during the mid-maturation stage, and then declines during late stages of seed development [9,10]. In the case of β-conglycinin, the accumulation of the β-subunit occurs much later than that of α′- and α-subunits during the soybean seed development [9,11]. Both the 7S and 11S globulins are initially synthesized on the rough ER, a membrane system consisting of an extensive, interconnected network of tubules and cisternae [12]. A N-terminal signal peptide present in the nascent polypeptide facilitates their entry into the ER. The correct folding and oligomerization of the storage proteins within the ER is aided by luminal chaperones and enzymes [13,14]. Additionally, the 7S globulins are cotranslationally glycosylated within the ER. Oligomers of the 7S and 11S globulins are transported to vacuoles by progression through the Golgi complex, before entering secretory vesicles that form PSVs [15].

The 7S and 11S globulins are deposited within specialized organelles called protein storage vacuoles (PSVs) in the cotyledonary parenchyma cells. Seed proteins that are targeted to PSVs contain vacuolar sorting determinants (VSDs). These VSDs can be sequence-specific (ssVSDs), C-terminal specific (ctVSDs), and physical structure-specific (psVSDs) [16]. In the case of soybean 7S globulins, the ctVSDs have been shown to be essential for sorting to the PSV. Interestingly, all three VSDs has been documented in soybean 11S globulins [17,18]. The PSVs are unique and can be differentiated from the lytic vacuoles (LVs) based on the type of tonoplast intrinsic proteins (TIPs) present in their membranes [19,20,21]. The membranes of PSVs are marked by α-TIP plus δ-TIP, while those of LVs are marked by γ-TIP [19,20,21]. Discrete distributions of different types of proteins within the PSVs and protein bodies (PB) have been reported in several plants [15,22]. In contrast, subdomains that contain different kinds of proteins within the PSVs have not been reported in soybean. Ultrastructural studies have revealed that soybean PSVs are filled with densely packed protein deposits that are uniformly amorphous [23,24]. Thus, it appears that both the 7S and 11S globulins of soybean are distributed evenly within the PSVs, giving an amorphous appearance. Interestingly, there is considerable solubility difference between 7S and 11S globulins, especially at acidic pH, so differential accretion of these proteins within the PSVs will not be surprising. Here, we employed immunocytochemical localization and confocal fluorescent microscopy to examine differential patterns of protein accretion and to determine the presence or absence of subdomains within the soybean PSVs.

## 2. Results

### 2.1. Light and Transmission Electron Microscopy of Soybean Seed Cotyledons

**Figure 1:**
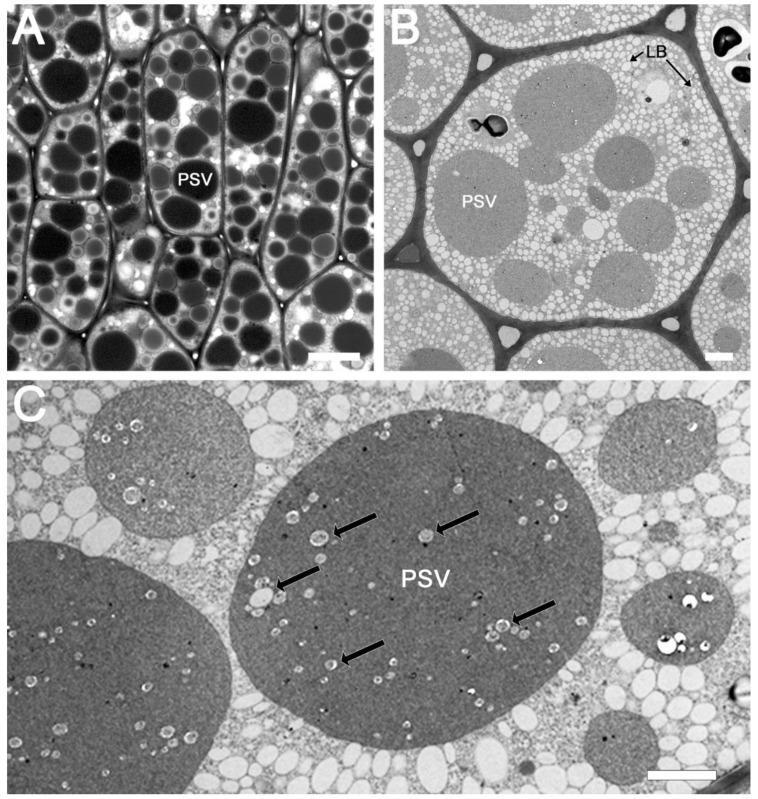
Visualization of protein storage bodies in the soybean seed cotyledon embedded in Spurr’s low-viscosity media using different microscopy techniques. Transmitted light image of toluidine blue-stained semi-thin section with 20 µm scale bar (**A**), and transmission electron microscopy images of soybean seed tissue dehydrated with acetone (**B**) or ethanol (**C**), with 2 µm scale bar. PSV = protein storage vacuole, LB = lipid body. Black arrows on image C point to phytate crystals.

The ultrastructure of soybean cotyledons has been examined by several investigators [23,24,25,26,27,28]. These studies have shown that soybean cotyledonary cells are filled with two major storage organelles, namely lipid bodies and protein storage vacuoles. To investigate the existence of subdomains within the protein storage vacuoles, we first examined sections of glutaraldehyde-fixed soybean cotyledons embedded in Spurr’s resin under light and electron microscopy. Light microcopy observation revealed the presence of numerous, mostly spherical shaped, protein storage vacuoles of different sizes within each cotyledonary cell (Figure 1A). The morphology of these PSVs was similar, with dark staining, densely packed inclusions. Similarly, transmission electron microscopy observation of thin sections of soybean cotyledons revealed numerous lipid bodies and several prominent PSVs. The lipid bodies, which store oil, appeared as small white spherical bodies and were distributed throughout the cell cytoplasm (Figure 1B). The PSVs were filled with densely packed amorphous protein deposits along with small phytate crystal inclusions (Figure 1C). The presence of potential subdomains within PSVs for the storage of 7S and 11S globulins was not discernable by these techniques.

### 2.2. Eosin Staining of Paraffin Embedded Soybean Cotyledons Facilitates in Depth Observation of Protein Storage Vacuoles by Confocal Fluorescent Microscopy

**Figure 2:**
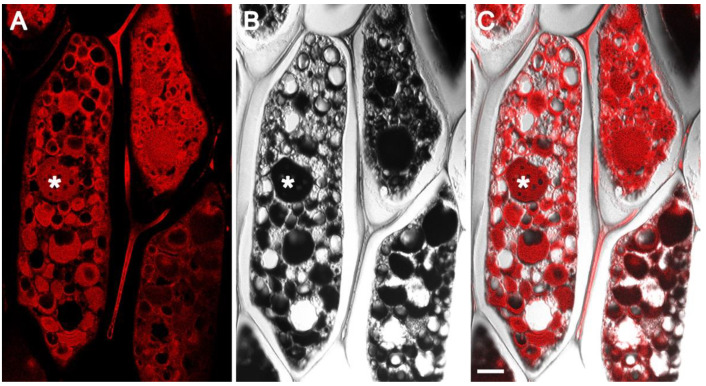
Confocal images of FAA-fixed, paraffin-embedded sections of the mature soybean cotyledon stained with eosin. Confocal single optical slice (**A**), DIC image (**B**), and DIC and single optical slice overlay (**C**). Asterisk indicates protein storage vacuole. Scale bar 10 µm.

Fluorescence microscopy of hematoxylin and eosin (H&E) stained sections have been widely used in animal and human studies for examining morphological and structural aspects of healthy and pathological tissue [29]. Hematoxylin, which stains nuclear components, has practically no fluorescence emission. Eosin is an acidic dye that binds to positively charged amino groups of proteins, staining them pink. Moreover, eosin is more commonly used as a chromogenic dye; it has fluorescent properties with an excitation peak at 525 nm and an emission peak at 546 nm. In this study, we made use of eosin fluorescence for examining protein storage vacuoles in soybean cotyledons by laser scanning confocal microscopy. Formaldehyde-alcohol-acetic acid (FAA) fixed, paraffin-embedded soybean seed sections, when viewed by confocal microscopy, revealed several protein storage vacuoles of various sizes inside individual cells (Figure 2). Interestingly, confocal microscopy observation showed the presence of intricate features within the PSVs (Figure 2A,B). However, these domains were not observed in complementary differential interference contrast (DIC) images (Figure 2B).

### 2.3. Confocal Fluorescent Microscopy Reveals the Presence of Potential Subdomains within Soybean Protein Storage Vacuoles

**Figure 3:**
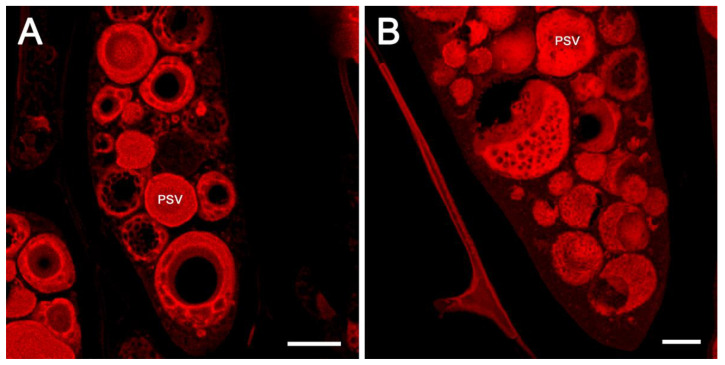
High-magnification confocal images of protein storage vacuoles stained with eosin. A cross-section of soybean cotyledons revealing apparent subdomains in the protein storage vacuoles with a scale bar of 10 µm (**A**). Uniformly spaced cavities on the surface of the PSV with a scale bar of 5 µm (**B**). PSV = protein storage vacuole.

Earlier studies have shown the existence of subdomains within PSVs that accumulate different kinds of proteins. We wanted to examine if similar subdomains were also present in soybean seed PSVs. We speculated that the two most abundant seed proteins of soybean, 7S β-conglycinin and 11S glycinins, which exhibit considerable solubility differences and temporal accumulation patterns, may be stored in distinct regions within the PSVs. Confocal fluorescent microscopy observation of FAA-fixed soybean seeds showed the presence of potential subdomains within the PSVs (Figure 3A,B). The PSVs in some cells were filled with protein inclusions. However, proteins occupying the central region of the PSVs were often lost, and the PSVs appeared as hollow structures (Figure 3A). Additionally, the outer regions of these PSVs also contained several small hollow regions (Figure 3A). The soybean seed PSVs resembled perforated spheres when examined by confocal fluorescent microscopy (Figure 3B).

### 2.4. Confocal Fluorescent Microscopy Localization of 7S and 11S Globulins Does Not Support the Existence of Subdomains within the Soybean Protein Storage Vacuoles

**Figure 4:**
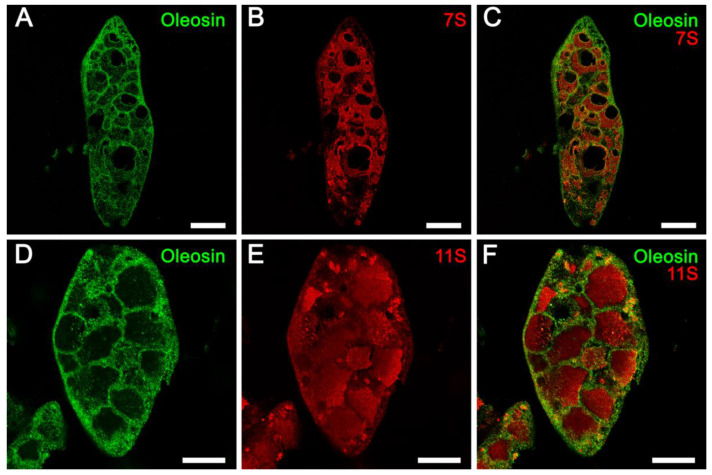
Fluorescence immunolabeling of oleosin, 7S and 11S globulins. Specific binding of the antibodies was detected by incubating the sections with either Alexa Fluor 488 labeled goat anti-chicken IgY antibodies (oleosin) or with Alexa Fluor 594 labeled goat anti-rabbit IgG antibodies (7s or 11S). PSV = protein storage vacuole, LB = lipid body. Scale bars for (**A**–**C**) are 20 µm. Scale bars for (**D**–**F**) are 10 µm.

Immunofluorescence localization of abundant soybean seed proteins and oleosin was performed utilizing antibodies raised against the respective purified proteins. Previous studies have shown that both the 7S and 11S globulins are localized within PSVs, while the oleosins are localized in oil bodies [25,26]. To simultaneously localize both the oleosins and globulins, antibodies to soybean 7S β-conglycinin or soybean 11S glycinin (raised in rabbits) and rapeseed oleosin antibodies (raised in chickens) were used. Alexa Fluor 594 labeled goat anti-rabbit IgG antibodies and Alexa Fluor 488 labeled goat anti-chicken antibodies were utilized as secondary antibodies. Observation of paraffin-embedded soybean seed cotyledons incubated with oleosin antibody and green-fluorescent dye (Alexa Fluor 488) labeled secondary antibodies clearly showed intense green fluorescence associated with numerous spherical particles that were distributed in the cell cytoplasm (Figure 4A,D). As expected, no green fluorescence was associated with the PSVs (Figure 4A,D). In contrast, when the same sections were incubated either with 7S or 11S globulin antibodies generated in rabbits and red-fluorescent dye (Alexa Fluor 594) labeled secondary antibodies, intense red fluorescence was detected on the PSVs (Figure 4B,E). The clear localization of the 7S and 11S globulins and oleosin became evident when these images were superimposed (Figure 4C,F). Importantly, both the 7S and 11S globulins were localized uniformly throughout the PSVs. Thus, our fluorescence immunolabeling study did not support the assumption that these two abundant groups of proteins are segregated into subdomains within the PSVs (Figure 4).

### 2.5. Subdomains within Soybean Protein Storage Vacuoles: A Reflection of In Situ Situation or a Fixation Artifact?

**Figure 5:**
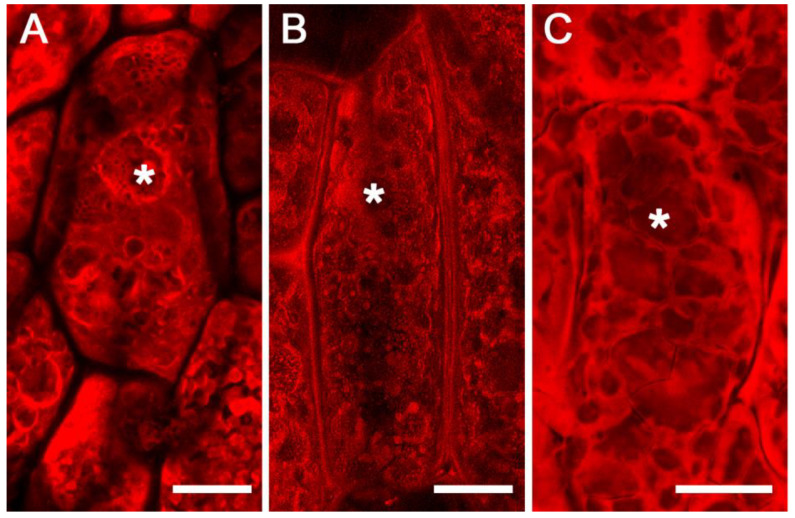
Maximum projection confocal images of eosin-stained soybean cotyledons. Free-hand section (**A**), vibratome section (**B**), and cryostat section (**C**). Asterisks indicate protein storage vacuoles. Scale bars for (**A**–**C**) are 20 µm.

Since our fluorescence immunolabeling studies did not support the existence of subdomains within the PSVs, we wanted to examine if the apparent subdomains that were visible in FAA-fixed soybean cotyledons could be due to a fixation artifact. To investigate this possibility, we carried out fixation-independent procedures. In the first approach, we examined free-hand sections of soybean cotyledons stained with eosin by confocal microscopy. Interestingly, most of the PSVs appeared to be filled with amorphous protein inclusions with no evidence of subdomains. However, the presence of sporadic subdomains was evident in a few PSVs (Figure 5A). Similarly, we also used a vibratome to obtain thick sections of fresh soybean cotyledons. Examination of vibratome sections revealed no apparent subdomains within the PSVs (Figure 5B) However, the protein inclusions appeared to be washed out, as evidenced by the presence of empty spaces within the PSVs (Figure 5B). Similarly, we also examined eosin-stained cryosections and found PSVs that were filled with amorphous protein inclusions (Figure 5C). Unlike FAA-fixed material, no elaborate internal subdomains were evident in cryosections (Figure 5C).

**Figure 6:**
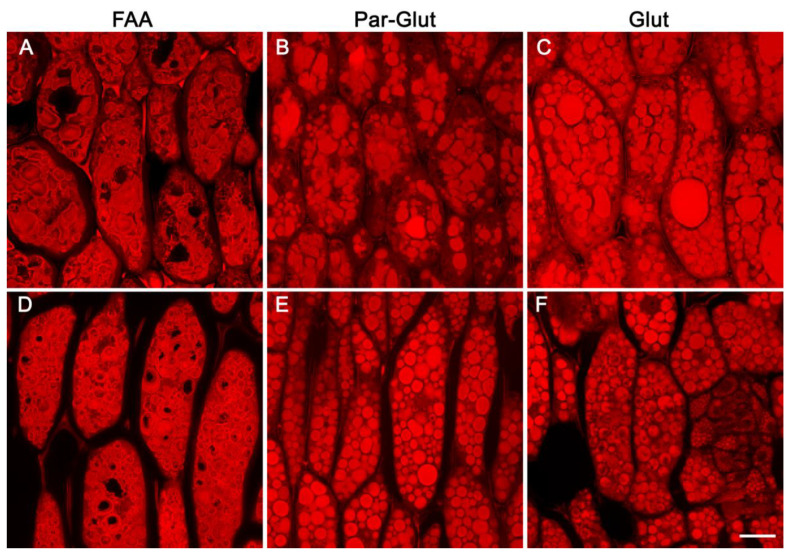
Confocal images of paraffin-embedded sections of the mature soybean cotyledon stained with eosin. (**A**,**D**)–fixed in FAA, (**B**,**E**)–fixed in 2% paraformaldehyde and 2% glutaraldehyde, (**C**,**F**)–fixed in 2.5% glutaraldehyde. Pictures on the top panel represent mature seeds, and those in the lower panel denote developing seeds at R6 stage. Scale bar in panel F is 20 µm and represents the scale for all panels.

Confocal scanning microscopy examination of fixation-independent soybean seed tissues indicated the apparent subdomains observed in FAA-fixed seeds may be a fixation artifact. Hence, we investigated the effect of three different fixatives on the appearance of PSVs. For this experiment, we used both developing and one-day-after-germination seeds. As observed earlier (Figure 3), FAA-fixed soybean seed sections revealed subdomains within the PSVs (Figure 6A,D). Interestingly, subdomains were much more conspicuous in one-day-after-germination seeds compared to developing seeds (Figure 6A,D). In direct contrast, examination of glutaraldehyde/paraformaldehyde- or glutaraldehyde-fixed seed material revealed that the PSVs were completely filled and revealed no apparent subdomains (Figure 6B,C,E,F).

## 3. Discussion

Previous ultrastructural analysis of soybean seed has shown that 7S and 11S globulins are deposited inside the PSVs. These two groups of abundant seed proteins are thought to co-exist as amorphous deposits with no obvious subdomains within the PSVs. In several plant species, PSVs are differentiated into subdomains that contain different kinds of proteins. In general, three morphologically distinct regions—the matrix, crystalloid, and globoid—can be observed in PSVs [30,31]. The matrix and crystalloid contain storage proteins, whereas the globoid represents phytic acid crystals [30,31]. The occurrence of subdomains has been reported in protein bodies (PBs) and PSVs of dicot and monocot seeds [15,22]. In rice endosperm, immunogold localization studies have shown that different classes of storage proteins are localized in discrete regions within the PSVs [32]. Rice glutelins were localized in the matrix, while the globulins were segregated in discrete zones within the PSVs [33]. In the case of pumpkin, three morphologically distinct regions—the matrix, crystalloid, and globoid—have been reported within PSVs. Immunocytochemical localization has demonstrated that the 7S proteins are in the peripheral matrix, while the 11S proteins are the primary constituent of the crystalloids [34]. However, no such morphologically distinct regions are seen in soybean PSVs, and both the 7S and 11S globulins appear to coexist as amorphous inclusions.

The confocal scanning microscope has been widely used for the investigation of subcellular structure, particularly of fluorescently stained tissue sections [35]. The fluorescent properties of eosin and its ability to bind to most proteins enabled us to use this stain for in-depth visualization of PSVs in soybean seeds. In our current study, we found that FAA-fixed paraffin-embedded soybean seed sections, when viewed by confocal fluorescence microscopy, revealed the occurrence of potential subdomains within PSVs. This observation indicated that seed proteins in the PSV were self-segregating into domains within the PSVs. The self-aggregation could be due to the considerable solubility and protein chemistry differences between 7S and 11S proteins. These differences could promote electrostatic interactions that would favor the association of some protein partners over others. Another possibility was that protein vesicles deposited into PSVs after exposure to acidic pH would be subjected to isoelectric precipitation that would favor accretions enriched in specific protein species. Even though confocal fluorescent microscopy observation of FAA-fixed eosin-stained soybean seed sections gave the impression that there are subdomains within the PSV, this is likely a fixation artifact. The fact that such subdomains within the PSV are not seen in seed sections prepared from fixation-independent procedures does not support differential patterns of protein accretion producing subdomains in the PSVs. Similarly, subdomains were not seen when the soybean seeds were fixed either in glutaraldehyde/paraformaldehyde or glutaraldehyde, indicating the apparent subdomains seen in FAA-fixed seed tissue were a fixation artifact.

The classic plant fixative FAA enables chemical cross-linking of cellular constituents, resulting in preservation of cell structure. Alcohol, the other component in the fixative, removes water from tissues, leading to coagulation and denaturation of proteins. However, it lacks mordant effect and could lead to retraction in the tissue. Acetic acid, which causes a change in the colloidal state of proteins, is used to counter the shrinkage caused by ethanol. In contrast to FAA, the two other fixatives employed in this study (2.5% glutaraldehyde in 100 mM cacodylate buffer pH 7.2, or 2% paraformaldehyde and 2% glutaraldehyde in 100 mM cacodylate buffer pH 7.2) are non-coagulating fixatives that chemically cross-link cellular constituents, allowing for preservation of structure. Thus, the marked differences in the appearance of soybean PSVs when viewed by confocal fluorescence microscopy may be due to the differences in the chemical interaction of the fixatives with the soybean seed cellular constituents.

## 4. Conclusions

In this study, we have demonstrated that fluorescent properties of the common protein-binding dye eosin, combined with three-dimensional laser scanning confocal microscopy, can be successfully used to observe the structure of PSVs in eosin-stained sections of soybean seeds. Additionally, our findings demonstrate the importance of appropriate choice of a fixative for studying the morphology of PSVs. Our observations suggest that FAA, a widely used fixative in histological studies of plant tissues, may induce artifacts that alter the appearance of the PSVs and give a false impression of the existence of subdomains within the soybean PSVs. Alternative fixatives such as glutaraldehyde/paraformaldehyde may be employed to minimize fixation-induced artifacts. 

## 5. Materials and Methods

### 5.1. Plant Materials

Soybean cultivar Williams 82 was used in this study. Seeds of Williams 82 were individually grown in 2-gallon pots containing PRO-MIX medium (Premier Horticulture, Quebec City, QC, Canada) in an environmentally controlled greenhouse. Soybean plants were fertilized three times during the growing period with Osmocote Plus (Scotts, Marysville, OH, USA). Developing seeds at R-6 and R-7 stages [36] as well as mature dry seeds harvested from these plants were used for anatomical investigation.

### 5.2. Tissue Fixation for Light Microscopy

Mature dry seeds of Williams 82 were first imbibed in water for 10 min, transferred to 1% agar plates, and allowed to germinate for 16 h in an incubator set to 30 °C. Following this, the seeds were sliced into several pieces with a razor and immediately fixed in 50% ethanol, 5% glacial acetic acid, and 10% formaldehyde (FAA). In addition to germinating seeds, developing seeds at R-6 and R-7 stage were also separately fixed in FAA for light microscopy. Additionally, seed samples were also separately fixed either in 2.5% glutaraldehyde in 100 mM cacodylate buffer, pH 7.2, or 2% paraformaldehyde and 2% glutaraldehyde in 100 mM cacodylate buffer, pH 7.2.

Fixed seed samples were dehydrated with an increasing ethanol series (80–95–100%), then with xylene, and finally immersed in liquid paraffin, using the Sakura Tissue-Tek VIP 5 (Sakura Finetek USA, Inc., Torrance, CA, USA). The samples were embedded in paraffin blocks using a Tissue-Tek Tissue Embedding Console System (Sakura Finetek, Torrance, CA, USA). Five-μm-thick sections were cut with an HM355 microtome (Thermo Fisher Scientific, Kalamazoo, MI, USA), collected on slides and placed in an oven at 60 °C for 30 min. The slides were then deparaffinized with xylene, then hydrated with ethanol and distilled water. Both the deparaffinization and hydration processes were performed by a Leica ST5020 Autostainer (Leica Biosystem; Buffalo Grove, IL, USA). Sections were then stained with eosin Y, coverslipped, and imaged using a laser scanning confocal microscope with a 63x/NA 1.4 oil immersion objective as described below.

### 5.3. Hand-Cut, Vibratome and Cryostat Sections

Germinated seeds were cut by hand with a razor blade at a variable thickness of approximately 150–300 µm, or with a vibratome (Vibratome 3000 Plus, Leica Microsystems Inc., Buffalo Grove, IL, USA) at a thickness of 100 µm. Sections were stained in 0.1% aqueous solution of eosin Y, mounted on #1.5 glass coverslips, and imaged with a Leica SP8 laser scanning confocal microscope (Leica Microsystems, Buffalo Grove, IL, USA) equipped with a tunable supercontinuum white light laser and a 63×/NA1.20 water immersion objective. Eosin fluorescence was excited with the 530 nm wavelength, and the emission was recorded using a 540–590 nm bandwidth. A series of images (z-stacks), placed at regular intervals of 0.8–1 µm, were acquired and used to generate maximum projection images.

### 5.4. Light and Transmission Electron Microscopy

Soybean seeds were cut into small pieces (2–4 mm cubes) and fixed for 4 h in 2.5% glutaraldehyde in 100 mM cacodylic buffer, pH 7.2. They were processed and embedded in Spurr’s low-viscosity media as described earlier [37].

### 5.5. Immunofluorescence Histochemistry

Immunohistochemical analysis was performed on paraffin sections using antibodies raised in rabbits against purified soybean 7S and 11S globulins [38,39]. Antibodies raised in mouse against purified *Brassica napus* seed oleosin [40] were also employed in this study. Five-µm sections of paraffin-embedded soybean cotyledons mounted onto X-tra Plus microscope slides (Leica, Richmond, IL, USA) were de-waxed in xylene, then rehydrated through graded concentrations of ethanol, and finally in water. Sections were then incubated for 60 min at room temperature with 1:200 dilution of a *Brassica napus* seed oleosin antibody followed by a 30 min incubation with Alexa Fluor 488 Plus-conjugated goat anti-chicken IgY secondary antibody (Invitrogen/ThermoFisher, Waltham, MA, USA) diluted 1:500. Following this step, the sections were washed and incubated with 1:200 dilution of a rabbit soybean 7S β-conglycinin antibody or 11S glycinin antibody for 60 min at room temperature. Sections were then washed and incubated for 30 min with 1:500 diluted Alexa Fluor 594 Plus-conjugated goat anti-rabbit secondary antibody (Invitrogen/ThermoFisher, Waltham, MA, USA). Sections were coverslipped with a mounting medium containing an antifade and observed under a Leica SP8 laser scanning confocal microscope (Leica Microsystems, Buffalo Grove, IL, USA) with a 20x/NA 0.7 objective using a 495 nm excitation laser line and a 505–550 nm bandpass or 550 nm excitation laser line and a 562–612 nm bandpass.

## Data Availability

Not applicable.

## Abbreviations

PSV	Protein storage vacuole
TEM	Transmission electron microscope
H&E	Hematoxylin and Eosin
FAA	Formaldehyde -Alcohol -Acetic Acid
TIP	Tonoplast intrinsic proteins
VCD	Vacuolar sorting determinant
